# Postoperative Diagnosis of Miliary Tuberculosis Masquerading as Acute Surgical Abdomen in an Immunocompromised Child: A Case Report

**DOI:** 10.7759/cureus.109477

**Published:** 2026-05-23

**Authors:** Hamza Talbi, Hicham Ziani, Anas El Hanbali, Leila Drouzi, Zakariya Hamdani, Mahmoud Byadi, Noussaiba Nabil, Saad Elharrak, Meryem Ennafiri, Larbi Dafali, Aziza Bentalha, Alae El Koraichi, Salma Ech Cherif El Kettani

**Affiliations:** 1 Pediatric Intensive Care Unit, Faculty of Medicine and Pharmacy, Mohammed V University, Rabat, MAR; 2 Department of Pediatric Anesthesiology, Faculty of Medicine and Pharmacy, Mohammed V University, Rabat, MAR

**Keywords:** case report, immunosuppression, juvenile idiopathic arthritis, miliary tuberculosis, postoperative complications

## Abstract

Miliary tuberculosis (TB) is a severe, life-threatening form of disseminated disease. In the postoperative setting, particularly among immunocompromised patients, its diagnosis is frequently delayed because its non-specific clinical presentation can easily masquerade as routine surgical complications.

We report the case of an eight-year-old female with juvenile idiopathic arthritis, managed with chronic immunosuppressive therapy (methotrexate and adalimumab), who presented with acute abdominal symptoms. Over a one-month period, she underwent three major surgical interventions for a suspected appendiceal abscess and subsequent adhesive bowel obstruction, complicated by multiple intra-abdominal abscesses. In the immediate postoperative period following her third surgery, she developed severe acute respiratory distress. High-resolution computed tomography revealed a diffuse bilateral micronodular pattern alongside an ileocecal phlegmon. An interferon-gamma release assay (IGRA) was positive. Rather than relying on a single test, the diagnosis of active miliary TB was established based on the compelling overall clinicoradiologic picture, specifically the setting of severe immunosuppression, the characteristic diffuse pulmonary and intra-abdominal findings, and the patient's positive response to targeted therapy. Following prompt admission to the intensive care unit and the initiation of first-line antitubercular therapy combined with broad-spectrum antibiotics and respiratory support, the patient achieved a full clinical recovery.

This case highlights the critical necessity for heightened diagnostic vigilance. Miliary TB must be included in the differential diagnosis of unexplained postoperative systemic failure or respiratory distress, particularly in immunocompromised patients or those originating from endemic regions. Early thoracic imaging and rapid therapeutic intervention are crucial determinants of patient survival.

## Introduction

Miliary tuberculosis is a rare and severe form of disseminated disease. The term 'miliary' derives from the characteristic radiologic and pathological appearance of the lesions, which resemble tiny millet seeds (typically 1 to 2 millimeters in diameter) uniformly distributed throughout the lungs and other organs. It results from the massive hematogenous dissemination of *Mycobacterium tuberculosis*, leading to multivisceral involvement. While the global burden of tuberculosis remains high, particularly in endemic regions where latent infections are prevalent, in pediatrics, miliary TB remains associated with high morbidity due to immune system immaturity and a frequently nonspecific clinical presentation, which can result in diagnostic delays.

The occurrence of miliary tuberculosis in the postoperative period is exceptional, particularly in children. However, surgical stress and transient immune disturbances induced by major surgery may promote the reactivation or dissemination of latent tuberculosis infection. Children receiving biologic immunosuppression, particularly tumor necrosis factor-alpha (TNF-α) inhibitors, such as adalimumab, are at an especially high risk. These agents disrupt granuloma formation and maintenance, thereby removing the host's primary defense mechanism and facilitating rapid mycobacterial reactivation and dissemination.

Distinguishing abdominal or disseminated tuberculosis from common surgical pathology presents a profound diagnostic challenge. Extrapulmonary abdominal tuberculosis can perfectly mimic routine surgical emergencies such as an appendiceal abscess or adhesive bowel obstruction. Furthermore, unlike routine postoperative bacterial infections that typically present with purulence and respond rapidly to broad-spectrum antibiotics, miliary TB may manifest as unexplained, sterile inflammatory masses or progressive postoperative systemic failure.

This case illustrates this exact diagnostic peril: the delayed recognition of disseminated miliary TB in a chronically immunosuppressed host, wherein abdominal extrapulmonary TB initially masqueraded as an acute surgical process. We report the case of an eight-year-old girl on immunosuppressive therapy who was diagnosed with miliary tuberculosis in the postoperative course of an acute abdomen. By detailing this unusual presentation, we aim to highlight how surgical unmasking and diagnostic mimicry can complicate the clinical picture, emphasizing the need for a high index of suspicion in immunocompromised pediatric patients, especially those with potential exposure in endemic settings.

## Case presentation

We report the case of an 8-year-old female weighing 22 kg. She had a history of juvenile idiopathic arthritis since the age of 4, which is treated with chronic immunosuppressive therapy (methotrexate and adalimumab). The patient had received the Bacillus Calmette-Guérin (BCG) vaccine. However, screening for latent tuberculosis infection (LTBI), including tuberculin skin testing (TST) or an interferon-gamma release assay (IGRA) and baseline chest imaging, was not performed prior to the initiation of biologic therapy. Furthermore, clinical history revealed documented exposure to a contact with active tuberculosis, yet no LTBI prophylaxis had been administered. Her condition was complicated by bilateral uveitis and a right-eye cataract, which was surgically treated three years ago.

The patient initially presented to the pediatric surgical emergency department with right lower quadrant (RLQ) abdominal pain, postprandial vomiting, and fever. Laboratory investigations revealed prominent leukocytosis. A preoperative abdominal ultrasound demonstrated clustered bowel loops in the right iliac fossa (RIF); notably, no computed tomography (CT) imaging was obtained prior to this first intervention.

Empirical broad-spectrum intravenous antibiotic therapy comprising a third-generation cephalosporin, an aminoglycoside, and metronidazole was initiated. Due to a high clinical suspicion of an appendiceal abscess, she underwent an exploratory laparotomy. Intraoperative findings revealed a dense, complex inflammatory mass without purulent exudate. Due to the difficult dissection and high risk of iatrogenic injury, definitive surgical resection was deferred. During this procedure, a sample of peritoneal fluid was collected and sent for routine histopathological examination. However, because abdominal tuberculosis was not yet considered in the differential diagnosis despite the patient's immunosuppressed state, no tissue, pus, or fluid samples were sent for Ziehl-Neelsen staining, mycobacterial culture, or nucleic acid amplification testing (NAAT/PCR).

Her postoperative course was unfavorable, characterized by persistent plateau fever and ongoing abdominal guarding despite seven days of targeted antibiotic therapy. Consequently, a second surgical intervention was performed. During this re-exploration, the previously noted dense inflammatory mass had partially resolved following the antibiotic course, which facilitated safer tissue dissection. An appendectomy was then successfully completed. The patient was subsequently discharged following an initially favorable clinical trajectory.

Five days post-discharge, the patient was readmitted with signs of acute intestinal obstruction, including severe abdominal pain, bilious vomiting, and absolute cessation of bowel movements and flatus. A plain abdominal radiograph showed multiple air-fluid levels, and an abdominal ultrasound identified a RIF abscess collection with moderate interloop fluid effusion.

An emergency laparotomy for suspected adhesive bowel obstruction was performed. Intraoperative exploration revealed extensive pathology, including multiple celiomesenteric and lumbo-aortic abscess collections, alongside a profound ileocecal phlegmon.

During the immediate postoperative period following her third surgery, the patient developed acute respiratory distress, manifesting as severe tachypnea (50 breaths per minute) and profound hypoxemia (oxygen saturation of 70% on room air). Laboratory investigations revealed leukocytosis with elevated inflammatory markers.

**Figure 1:**
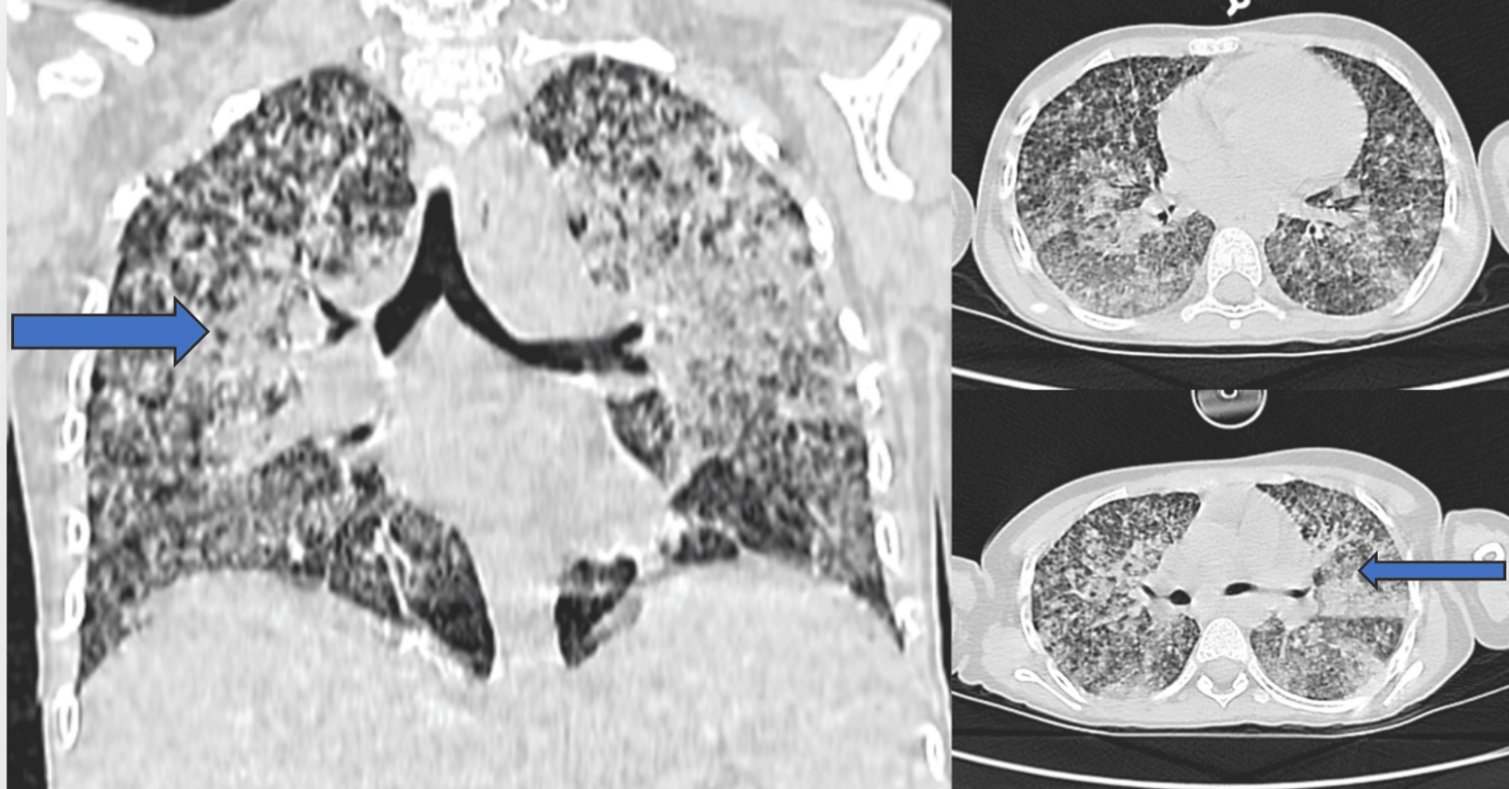
Chest CT scan showing a miliary tuberculosis pattern (left: coronal view; right: two axial views)

**Figure 2:**
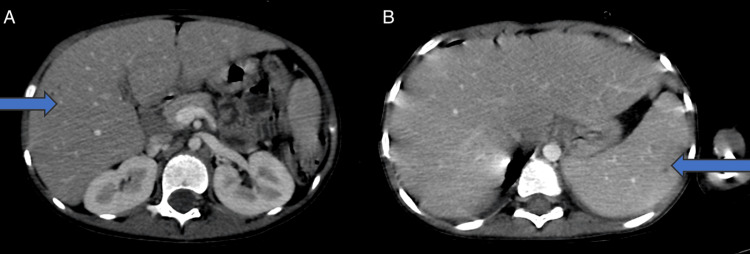
Axial views of an abdominal CT scan showing microabscesses in the liver (A) and spleen (B)

A chest radiograph revealed diffuse bilateral micronodular infiltrates. A subsequent thoracoabdominopelvic computed tomography (CT) scan confirmed a miliary pattern (Figure 1), accompanied by disseminated microabscesses in the liver and spleen (Figure 2), and corroborated the presence of the ileocecal phlegmon.

She was immediately transferred to the intensive care unit (ICU). Extensive infectious disease screening, including serologies for hepatitis B and C, human immunodeficiency virus (HIV), cytomegalovirus (CMV), Epstein-Barr virus (EBV), and Aspergillus, returned negative. An interferon-gamma release assay (IGRA; Lioferon TB/LTBI, Lionex GmbH, Braunschweig, Germany) was positive, confirming prior sensitization to *Mycobacterium tuberculosis* (Table 1). However, direct microbiologic confirmation of active disease (via cultures or PCR) was not obtained. Consequently, the diagnosis of active miliary tuberculosis was established presumptively, based on the compelling clinicoradiologic picture and the patient's favorable response to empirical antitubercular therapy*.*

**Table 1:** A positive interferon-gamma release assay (IGRA)

Parameter	Value (IU/mL)	Reference Cutoff (IU/mL)	Result
Negative Control (Nil)	0.140	-	-
TB Antigen A - Nil	2.18	< 0.35	Positive
TB Antigen B - Nil	2.22	< 0.35	Positive
Positive Control - Nil	7.53	-	-

Upon the suspicion of active miliary tuberculosis and in the setting of severe respiratory distress, both methotrexate and adalimumab were immediately discontinued. Systemic corticosteroids were not administered, and management focused solely on targeted antimicrobial therapy.

The patient was promptly started on first-line antitubercular therapy consisting of rifampicin, isoniazid, pyrazinamide, and ethambutol. Drug dosing was strictly optimized according to pediatric weight-based guidelines for her 22 kg body weight. Despite the initiation of anti-TB therapy, broad-spectrum antibiotic coverage using intravenous imipenem (500 mg every 8 hours) and amikacin (330 mg daily) was deliberately maintained. The rationale for continuing these antibiotics was the presence of the ileocecal phlegmon and suspected intra-abdominal abscesses, necessitating robust coverage for a concurrent superimposed bacterial peritonitis or intra-abdominal sepsis.

Given the known toxicities of the regimen, vigilant clinical and laboratory monitoring was instituted. Regular liver function tests were performed to monitor for hepatotoxicity induced by isoniazid, rifampicin, and pyrazinamide. Furthermore, owing to the patient's prior history of bilateral uveitis and cataract surgery, strict ophthalmologic monitoring was prioritized to rapidly detect any ethambutol-induced optic neuropathy. Respiratory support included daily non-invasive ventilation sessions combined with targeted respiratory physiotherapy.

The clinical evolution was highly favorable, marked by complete defervescence, weaning of oxygen therapy, and overall respiratory and hemodynamic stabilization. Following a one-week stay in the ICU, she was successfully transferred to the general pediatric ward for continued medical management. The total planned duration for the antitubercular treatment is nine months. At her latest outpatient follow-up, the patient remained clinically stable, with no signs of disease relapse.

## Discussion

Miliary tuberculosis is a severe form of tuberculosis resulting from the hematogenous dissemination of *Mycobacterium tuberculosis*, which can simultaneously affect multiple organs such as the lungs, liver, spleen, bone marrow, and central nervous system. This disseminated form is life-threatening in the absence of early diagnosis and appropriate treatment. It accounts for less than 2% of active tuberculosis cases but remains a medical emergency due to its rapid progression and high mortality, particularly among children, the elderly, and immunocompromised patients [1,2].

The pathophysiology of miliary tuberculosis is based on the hematogenous dissemination of the bacillus from a primary pulmonary or extrapulmonary focus, often occurring when cellular immune control fails. The rupture of a caseating granuloma into a blood vessel represents a key mechanism of this dissemination, releasing a large number of bacilli into the systemic circulation and leading to the formation of numerous microgranulomas in affected organs. This spread is facilitated by an inadequate immune response, particularly impairment of cell-mediated immunity driven by T lymphocytes [3].

Several risk factors have been identified in the literature. Miliary tuberculosis occurs more frequently in individuals with immunosuppression, whether related to viral infection (particularly HIV), immunosuppressive therapies (prolonged corticosteroid use, chemotherapy, biologic agents), or severe malnutrition. Immunosuppression promotes the systemic dissemination of the bacillus from an initial focus, which appears to correspond to the case of our patient [4].

In children, the risk of dissemination is particularly high due to the immaturity of the immune system, especially in infants and young children. The absence of Bacille Calmette-Guérin (BCG) vaccination or ineffective vaccination also constitutes a contributing factor. In tuberculosis-endemic areas, early exposure to the bacillus increases the risk of complicated primary infection, which may progress to miliary tuberculosis [5].

In the postoperative context, major surgery represents a significant physiological stress, associated with a systemic inflammatory response and transient immunosuppression. This immune alteration may promote the reactivation of latent tuberculosis or the dissemination of a subclinical focus. Several clinical observations have reported the occurrence of miliary tuberculosis following major surgical procedures, particularly when these lead to a catabolic state or malnutrition [6].

Cases have been reported following bariatric, digestive, or orthopedic surgery, in which rapid weight loss, nutritional deficiencies, and metabolic stress contribute to impaired immune defenses. These factors may promote the reactivation of latent tuberculosis infection, highlighting the importance of preoperative tuberculosis risk assessment in patients originating from endemic areas [7].

Exposure to tuberculosis and previous residence in high-prevalence settings, such as several countries in Africa or Asia, constitute major risk factors. In these contexts, latent tuberculosis infection (LTBI) is common and may remain asymptomatic for years before reactivating during periods of immunosuppression. In children, the absence of symptoms prior to surgery does not rule out the presence of a latent focus [8].

In the postoperative setting, the clinical presentation of miliary tuberculosis is often non-specific, characterized by prolonged fever, constitutional symptoms, anorexia, and weight loss. These signs may be erroneously attributed to normal surgical recovery, leading to diagnostic delays. Such delays are a critical determinant of poor prognosis, as mortality rates increase significantly with late diagnosis [9]. 

Autopsy studies have demonstrated that miliary tuberculosis can remain undetected until an advanced stage if not actively sought in patients with unexplained postoperative fever. Multisystemic dissemination may progress silently, particularly in immunocompromised patients, necessitating heightened clinical vigilance [10].

Furthermore, the literature highlights that the diagnostic yield of conventional examinations may be limited during the early stages of the disease. Chest X-rays may appear normal or non-specific, sputum smear microscopy is frequently negative, and immunological tests can yield false-negative results in cases of immunosuppression. In this context, additional investigations such as chest CT scans, molecular testing (PCR), and tissue biopsies may be required to confirm the diagnosis [11].

Thoracic imaging, particularly computed tomography (CT), plays a pivotal role in early diagnosis by detecting diffuse micronodules before they become visible on standard radiography. The presence of a bilateral diffuse micronodular pattern should primarily suggest miliary tuberculosis within a compatible clinical context [12].

In our patient’s case, several predisposing factors coalesced, including chronic immunosuppression with a TNF-alpha inhibitor, the potential for an unrecognized latent tuberculous focus in an endemic setting, and the physiological stress of three closely spaced surgical interventions. However, given the inherent limitations of a single case report, the exact pathophysiological sequence of events remains difficult to definitively establish. It is highly plausible that the profound immunosuppression induced by adalimumab was the principal driver of TB reactivation. Alternatively, the initial acute abdominal presentation may have actually represented unrecognized primary extrapulmonary (abdominal) tuberculosis mimicking a surgical emergency, with the subsequent surgical stress merely exacerbating the hematogenous dissemination. Acknowledging these multiple interacting variables and alternative explanations underscores the complexity of such cases and highlights the critical need for heightened diagnostic vigilance.

## Conclusions

Miliary tuberculosis is a severe, potentially fatal disease whose atypical postoperative presentation can easily be misattributed to conventional surgical complications, leading to dangerous diagnostic delays. To prevent such life-threatening scenarios, it is imperative to enforce rigorous pre-biologic and preoperative tuberculosis risk assessments, including comprehensive screening and exposure history, in pediatric patients receiving TNF-α inhibitors, especially in endemic regions. When such upstream preventative measures are absent or fail, this emphasizes the critical need to actively include miliary tuberculosis in the differential diagnosis of any unexplained postoperative systemic failure or acute abdominal emergency in chronically immunosuppressed hosts. Ultimately, early recognition and the rapid initiation of targeted antitubercular therapy remain the most crucial determinants of patient survival.
